# Missing Acknowledgement

**DOI:** 10.1001/jamahealthforum.2024.3480

**Published:** 2024-09-27

The Special Communication titled “Advancing Health Policy and Outcomes for People With Intellectual or Developmental Disabilities: A Community-Led Agenda,”^1^ has been updated to add an acknowledgement. Specifically, Rick Rader, MD, of the American Association on Health and Disability, participated in the consensus working group meetings that developed the draft national goals for the IIDDEAL project and reviewed the manuscript before submission. This article was corrected.
